# Low rate of infected knee replacements in a nationwide series—is it an underestimate? Review of the Finnish Arthroplasty Register on 38,676 operations performed in 1997 through 2003

**DOI:** 10.3109/17453670902947432

**Published:** 2009-04-01

**Authors:** Esa Jämsen, Kaisa Huotari, Heini Huhtala, Juha Nevalainen, Yrjö T Konttinen

**Affiliations:** ^1^Medical school, University of Tampere, TampereHelsinkiFinland; ^2^Coxa, Hospital for Joint Replacement, TampereHelsinkiFinland; ^3^Division of Infectious Diseases, Department of Medicine, Helsinki University Central Hospital, HelsinkiHelsinkiFinland; ^4^Tampere School of Public Health, University of Tampere, TampereHelsinkiFinland; ^5^Department of Applied Physics, University of Kuopio, KuopioHelsinkiFinland; ^6^Department of Medicine, Helsinki University Central HospitalHelsinkiFinland

## Abstract

**Background and purpose** Specialist hospitals have reported an incidence of early deep infections of < 1% following primary knee replacement. The purpose of this study was to estimate the infection rate in a nationwide series using register-based data.

**Methods** The Finnish Arthroplasty Register (FAR) was searched for primary unicompartmental, total, and revision knee arthroplasties performed in 1997 through 2003 and eventual revision arthroplasties. The FAR data on revision arthroplasties was supplemented by a search of the national Hospital Discharge Register (HDR) for debridements, partial and total revision knee replacements, resection arthroplasties, arthrodeses, and amputations.

**Results** During the first postoperative year, 0.33% (95% CI: 0.13–0.84), 0.52% (0.45–0.60) and 1.91% (1.40–2.61) of the primary UKAs, primary TKAs, and revision TKAs, respectively, were reoperated due to infection. The 1-year rate of reoperations due to infection remained constant in all arthroplasty groups over the observation period.

The overall infection rate calculated using FAR data only was 0.77% (95% CI: 0.69–0.86), which was lower, but was not, however, statistically significantly different from the overall infection rate calculated using endpoint data combined from FAR and HDR records (0.89%; 95% CI: 0.80–0.99). FAR registered revision arthroplasties and patellar resurfacing arthroplasties reliably but missed a considerable proportion of other reoperations.

**Interpretation** More reoperations performed due to infection can be expected as the numbers of knee arthroplasties increase, since there has been no improvement in the early infection rate. Finnish Arthroplasty Register data appear to underestimate the incidence of reoperations performed due to infection.

Recent studies have indicated that the incidence of deep infection after primary knee arthroplasty is below 1%. These figures were obtained from specialized institutions (Peersman et al. 2001, Phillips et al. 2006, Jämsen et al. 2008) and from the Finnish Hospital Infection Surveillance Program SIRO, in which results from 9 hospitals are drawn together (Huotari et al. 2006). Furnes et al. (2007) reported revision rates due to infection in the Norwegian Arthroplasty Register, but included only unicompartmental knee arthroplasties and tricompartmental cemented total knee arthroplasties. Thus, the sources and selection criteria used limit the generalizability of these results.

Scandinavian arthroplasty registers are generally considered to be reliable sources of data, and they are also considered to provide more realistic estimates of prosthesis survival and complication rates than case series from specialized institutions (Robertsson 2007). A shared feature of arthroplasty registers is that the event recorded is implantation or removal of one or more components of a joint prosthesis. Minor revision surgeries, resection arthroplasties, arthrodeses, and amputations—which are used in the management of deep periprosthetic infections (Leone and Hanssen 2006)—are poorly captured by arthroplasty registers. This may lead to underestimation of postoperative complication rates.

The purpose of this register-based study was to estimate the nationwide incidence of deep infections in contemporary unicondylar and total primary knee arthroplasty, and also revision total knee arthroplasty, and to determine how the infection rates changed during the period of rapid increase in annual numbers of knee arthroplasties in 1997–2003. To more reliably detect different types of septic reoperations, the records of the Finnish Arthroplasty Register were supplemented with the data of the national administrative Hospital Discharge Register.

**Figure 1.**
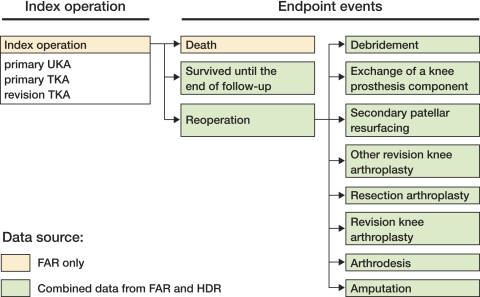
Flow chart showing the index operations, endpoint events, and the corresponding data sources.

## Material and methods

Data were obtained from the Finnish Arthroplasty Register (FAR) and the national Hospital Discharge Register (HDR). FAR, administered by the National Agency for Medicines, is based on mandatory reporting of joint replacement operations by surgeons (Puolakka et al. 2001, Rantanen et al. 2006). Unlike other Scandinavian arthroplasty registers, FAR has not been validated scientifically yet. However, FAR requires input on prosthesis components and attempts are made to retrieve missing information manually. Thus, it is likely that the operations and their descriptions as recorded by FAR are accurate.

The Finnish Hospital Discharge Register was founded in 1976 and is administered by the National Research and Development Center for Welfare and Health. It covers about 95% of all discharges from hospitals in Finland (Gissler and Haukka 2004). Data are collected with a hospitalization being the registration unit. For each period of hospitalization dates of admission and discharge, primary diagnosis and possible subsidiary diagnoses, surgical procedures performed, and type of hospital are recorded in the register in addition to demographic data. For classification of diagnoses, ICD-10 has been used since 1996. Since 1997, surgical procedures have been classified according to the Finnish (1997) version of the procedure classification of the Nordic Medico-Statistical Committee (NCSP-F, http://www.nordclass.uu.se/verksam/ncspe.htm). The accuracy of items recorded is considered good (Gissler and Haukka 2004), but validity concerning orthopedic diagnoses and procedures has not been assessed.

### Index operations

**Figure 2.**
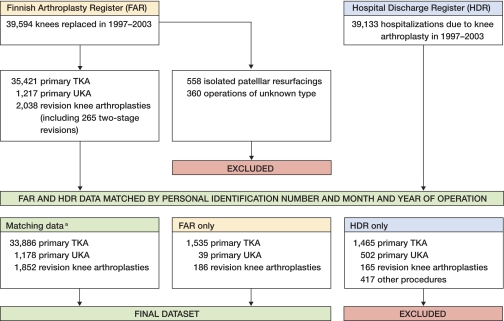
Flow chart indicating how the data from the Finnish Arthroplasty Register (FAR) and the Hospital Discharge Register (HDR) were combined to create the final dataset. **^a^**operation types according to FAR.

The Finnish Arthroplasty Register was searched to identify primary unicompartmental knee arthroplasties (UKAs), primary total knee arthroplasties (TKAs), and revision TKAs performed anywhere in Finland between January 1, 1997 and December 31, 2003. The operations that were selected to be followed up are referred to as index operations (Figure 1). Resection arthroplasties, partial exchange arthroplasties (secondary patellar resurfacing and/or isolated exchange of tibial insert), and operations of unknown type were excluded (Figure 2).

The final dataset (n = 38,676) consisted of 36,638 primary knee arthroplasties and 2,038 revision total knee arthroplasties (Figure 2). The FAR data on these operations could be supplemented by corresponding hospitalization data from HDR in 36,916 cases (95.4%). Primary operations could be matched to HDR data more frequently than revision TKAs (match in 95.7% of cases vs. 90.9%, p < 0.001). Because the validity of the data on knee arthroplasties registered by HDR but missing from FAR could not be confirmed, these operations were excluded as probable false entries (Figure 2).

### Endpoint data

Eventual revision knee arthroplasties (exchange or addition of one or more of the prosthesis components), other surgical reoperations, and deaths were considered endpoints of the follow-up (Figure 1). As we supposed that certain reoperations, including debridements, arthrodeses and amputations, are infrequently reported to FAR, data on endpoint events were also collected from HDR.

In FAR, endpoint data were readily available as all revisions are linked directly to the preceding arthroplasty. From HDR, endpoint data were collected using the Nordic Medico-Statistical Committee (NOMESCO) classification for surgical procedures (http://www.nordclass.uu.se/verksam/ncspe.htm). The Finnish version of the NOMESCO Classification of Surgical Procedures (NCSP-F) was introduced in 1997 and it was used unchanged over the observation period (1997–2003). The following surgical procedures were included (procedure codes according to the NCSP-F are presented in parentheses): revision knee arthroplasty for any reason (NGC20, NGC30, NGC40, NGC60, NGC99), removal of the prosthesis (NGU00), debridement (NGC00, NGF20, NGF25), amputation (NFQ20), and arthrodesis (NGG30, NGG34). Revision knee arthroplasties and other types of reoperations are later referred to collectively as reoperations.

Reoperations detected in either register were classified as being septic or aseptic. Reoperations where (1) FAR reported infection as the indication for reoperation, or (2) HDR reported an ICD-10 diagnosis code indicating arthroplasty-associated infection (T84.5, T81.4) or septic infection (in A series of codes), were considered septic. The remaining reoperations were considered aseptic. In 93% of cases, these 2 criteria were concordant. The remaining cases, where the registers did not agree about type of reoperation, were reviewed manually with the help of diagnosis and procedure codes and data from other operations involving the same joint.

Endpoint data were collected independently of FAR and HDR, and then the data of the 2 registers were combined. If 2 or more reoperations were registered for an index operation, the one performed first was used in the analyses. Overall, there were 1,658 reoperations following the 38,676 index operations under follow-up. 1,057 reoperations were classified similarly by the two registers. 313 reoperations were detected by HDR only and 177 by FAR only.

For most HDR endpoints (79%), a corresponding event in FAR could be detected and could therefore be reliably linked to the preceding index operation. If an endpoint event was registered in HDR only, we used the personal ID number along with the identity (i.e. side) of the operated knee (available in 47 of 313 cases) to link the endpoint to the latest index operation (supposing that complications requiring reoperation occur early rather than late). If in such a case the patient was known to have both knees operated, the follow-up of both knees was considered to end on the day of reoperation. No endpoint data were excluded from the analysis. If no reoperations were performed, the follow-up was considered to end at death or on December 31, 2004 (Figure 1).

### Statistics

The 1-year infection rate, defined as the number of reoperations performed due to infection within 1 year of the index arthroplasty per total number of operated joints, with its 95% confidence intervals (CIs) is reported as the primary outcome. Additionally, we present the overall infection rate (infections occurring within 1 year and those occurring later) and the average time from the index surgery until occurrence of an endpoint (presented as median and range). Only the first endpoint procedure following each index operation was taken into account when calculating the infection rates.

To determine whether the decisions made in the selection of materials and in defining the outcome had led to systematic bias in the results presented, we performed a sensitivity analysis (a so-called “what if” analysis). This was done by calculating the rate of infections in different patient subgroups, based on the source of data, with different criteria for reoperation due to infection. If the changes in patient selection and definition would not statistically significantly alter the initial results and would not lead to different interpretation, the results presented could be considered valid.

We used SPSS for Windows version 14.0 for all data management and statistical analyses. For statistical analysis, the hospitalization-level data of the HDR were converted to knee-level data by linking them to corresponding FAR data. Although the 2 knee prostheses in bilateral cases did not represent independent observations, we considered that due to the rarity of bilateral reoperations, ignoring bilaterality would not bias the results (Robertsson and Ranstam 2003) and all statistical analyses were performed with knee prosthesis as the statistical unit. Confidence intervals for infection rates were calculated using Wilson’s method. For comparison of infection rates, chi-squared test or Fisher’s exact test was used. We used binary logistic regression or two-way analysis of variance to show the significance of time trends. Survival curves with reoperation due to infection as endpoint were calculated using Kaplan-Meier survival analysis for primary UKA, primary TKA, and revision TKA. Probability values (p) of < 0.05 were considered statistically significant.

## Results

### Description of materials

Compared to patients undergoing primary TKA, those who underwent primary UKA were younger (64 vs. 71 years), had more osteoarthritis (98.8% vs. 90.2% of all patients), and were more often discharged home (80% vs. 68% of all patients) after a short hospitalization (6 vs. 9 days) (p < 0.001 for all comparisons) (Table 1). In the revision TKA group, diagnoses other than osteoarthritis were overrepresented (11.5% as compared to 9.8% in primary TKA, p = 0.002) and more patients were discharged to other healthcare institutions compared to primary TKA (40% vs 32%, p < 0.001). Most revision TKA operations were performed in university hospitals and central hospitals (68%). Loosening (25%), other reasons (24%; includes polyethylene wear) and infection (16%) were the most common indications for the index revision arthroplasties. The revision arthroplasties were performed on average 5.5 years (range 1 day to 29 years, 26% within 2 years) after the preceding operation.

**Table 1.** Patient demographics, perioperative details, and follow-up data for the 38,676 primary unicondylar arthoplasties (UKAs), total knee arthoplasties (TKAs), and revision total knee arthroplasties

	n or median	% or (range)
Age, years	71	(14–96)
Knees in female patients	28,043	73
Diagnosis		
Osteoarthritis	34,950	90
Rheumatoid arthritis	3,003	8
Other	723	2
Same-day bilateral arthroplasty	2,854	7
Cement fixation	35,516	92
Patellar resurfacing	12,150	32
Intravenous antibiotic prophylaxis	37,991	98
Antibiotic-impregnated cement	31,873	82
Any early complication	439	1.1
Operating hospital		
University hospital	8,405	22
Central hospital	12,999	33
District hospital	12,693	33
Other hospital	4,579	12
*Hospitalization ^a^*		
Arrival at hospital on the day of operation	4,185	11
Arrived from home	35,850	97
Length of stay in operating hospital, days	9	(1–106)
Discharged home	25,077	68
Referred for treatment from		
Primary healthcare	14,848	38
Private healthcare	10,192	26
Specialized healthcare	8,907	23
*Follow-up*		
Length of follow-up, months	41	(0–103)
Died during the follow-up	3,948	10

**^a^** Data on perioperative hospitalization were available in 36,916 cases.

The annual number of operations increased from 4,514 in 1997 to 7,552 in 2003. Over the observation period, the proportion of UKAs and of patients with OA among primary TKA recipients increased and the length of perioperative hospitalization decreased (Table 2). Antibiotic-impregnated cement was used in only 58% of cases in 1997 but in 97% of cases in 2003 (p < 0.001). The number of revision TKAs performed due to infection increased from 29 in 1997 to 67 in 2003. For revision TKA, the proportion of infections for all reasons showed some year-to-year variation but suggested a slight increase from 11% in 1997 to 20% in 2003 (p = 0.09).

**Table 2.** The changes in selected demographic and administrative variables over the observation period (1997–2003)

	Year of operation		p for
	1997	2003	trend
Number of operations	4,514	7,552	–
Type of operation			
primary UKA, %	1	5	< 0.001
primary TKA, %	93	90.0	< 0.001
revision TKA, %	6	5	< 0.001
Same-day bilateral procedures, %	5	8	< 0.001
Age at operation, average	71	71	< 0.001
age range	19–93	21–91	
Indication for primary TKA			
osteoarthritis, %	89	92	< 0.001
rheumatoid arthritis, %	10	6	< 0.001
Indication for revision TKA			
loosening, %	34	22	< 0.001
other reasons, %	24	24	0.6
infection, n	29	67	–
infection, %	11	20	0.09
Intravenous antibiotic prophylaxis, %	97	99	< 0.001
Antibiotic-impregnated bone cement, %	58	97	< 0.001
Days of hospitalization, median	10	7	< 0.001
range	1–106	1–48	
Arrival on the day of operation, %	2	20	< 0.001
Discharged directly home, %	69	63	< 0.001

UKA: unicondylar knee arthroplasty; TKA: total knee arthroplasty.

### Reoperations

**Figure 3.**
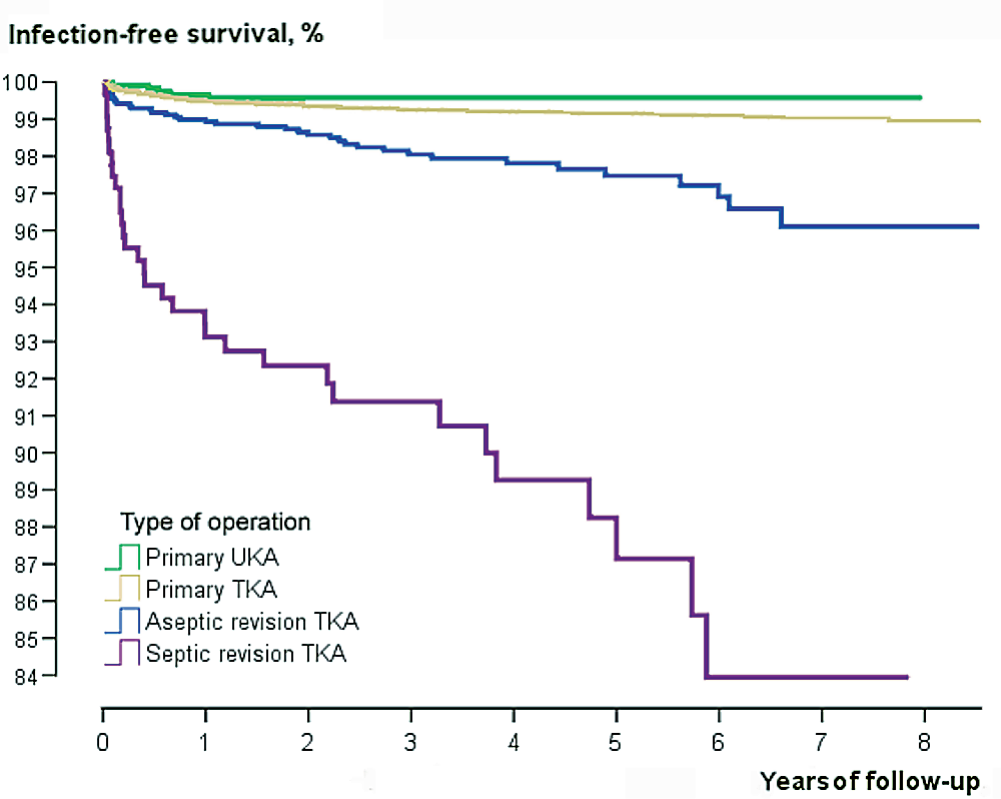
Prosthesis survival after primary unicompartmental arthroplasty (UKA), total knee arthroplasty (TKA), and for aseptic and septic revision TKA with any reoperation (including debridement, resection arthroplasty, partial or total revision arthroplasty, arthrodesis, or amputation) due to infection as endpoint. The data on the operations under follow-up were collected from the Finnish Arthroplasty Register (FAR). Endpoint data were collected from FAR and from the Hospital Discharge Register.

Of the 1,658 reoperations, 344 were due to infection (overall infection rate 0.89%, 95% CI: 0.80–0.99). The 1-year septic reoperation rates were 0.33% (95% CI: 0.13–0.84), 0.52% (0.45–0.60), and 1.91% (1.40–2.61) after primary UKA, primary TKA and revision TKA, respectively. The infection-free survival of the index revision TKAs performed for aseptic reasons was considerably better than survival of revision TKAs performed due to infection (Figure 3).

**Table 3.** The endpoint events of follow-up after 38,676 knee replacements. The endpoint events were traced from the records of the Finnish Arthroplasty Register (FAR), the Hospital Discharge Register (HDR), and by combining the data from these two registers (combined endpoint data). If two or more endpoint events were detected for an index operation, only the one occurring first was taken into account in the combined endpoint data

First endpoint event	In combined endpoint data	In FAR data		In HDR data	
	n	n	% **^a^**	n	% **^a^**
No endpoints registered	33,021	33,317	101	33,210	101
Died	3,948	4,014	102	3,985	101
Reoperation	1,658	1,345	81	1,481	89
debridement	18	0	0	18	100
resection arthroplasty	183	146	80	185	101
revision knee arthroplasty	541	496	92	559	103
secondary patellar resurfacing	303	259	85	303	100
exchange of a knee					
prosthesis component	168	131	78	170	101
other revision knee arthroplasty	195	79	41	196	101
arthrodesis	26	13	50	28	108
amputation	22	2	9	22	100
unknown to HDR	202	219	108	–	
Septic endpoint	344	298	87	295	86
Aseptic endpoint	1,314	1,047	80	1,186	90
Median length of follow-up,					
months (range)	41 (0–103)	42 (0–103)		42 (0–103)	

**^a^** The percentage value indicates the proportion of endpoints detected by FAR or HDR, of the number of such first endpoints in the combined endpoint data. Values of > 100% indicate that some of the reoperations were preceded by another type of reoperation detected in the other register (e.g. resection arthroplasty registered in FAR prior to arthrodesis registered in HDR).

After primary and revision TKA, 35% of all reoperations due to infection were performed during the first 3 postoperative months and approximately two-thirds took place within 1 year of the the index arthroplasty. Most infections after UKA (4/5) occurred during the first postoperative year (Figure 3). However, there were only 415 UKAs with follow-up exceeding 3 years.

The 1-year infection rates following primary UKA, primary TKA, and revision TKA remained constant from 1997 to 2003 (data not shown). The increase in the number of reoperations due to infection within each procedure and diagnosis group paralleled the increase in operation volume (not shown). Over the observation period, no changes in demographic or operative variables in those who underwent reoperation due to infection were seen (data not shown).

### Reoperations due to infection in FAR

Overall, FAR had registered 1,345 reoperations, 298 (22%) of which were performed due to infection. Thus, 46 reoperations due to infection (13%) would have remained undetected if only the records of FAR had been used. The overall infection rate calculated using FAR data only was 0.77% (95% CI: 0.69–0.86), which however, was not statistically significantly different from the overall infection rate calculated using combined endpoint data (0.89% (0.80–0.99)). In 225 cases, the FAR register entry for reoperation due to infection could be matched to the corresponding endpoint event (with appropriate infection diagnosis code) in the HDR data, leading to an overall infection rate of only 0.6% (0.51–0.66).

When compared with the endpoint data combined from the FAR and HDR records, FAR most successfully captured revision arthroplasties and secondary patellar resurfacings but failed to detect most other revisions (NGC99), arthrodeses, amputations, and debridements (Table 3).

Most of the reoperations due to infection that were detected only by HDR occurred during the first 3 postoperative months when most of the reoperations classified as partial exchange arthroplasties, other revision arthroplasties (NGC99), or amputations were also performed (the proportions of operations performed within 3 months were: 56%, 46%, and 67%, respectively). On the other hand, 46% of septic reoperations recorded by FAR only were performed during the first 3 postoperative months.

### Sensitivity analyses

In the sensitivity analyses overall infection rates were calculated using different datasets (all operations registered to HDR; operations registered similarly in FAR and HDR; operations registered in either FAR or HDR but missing from the other register; all operations registered in either of the two registers) in addition to the final dataset used in the analyses (all operations registered to FAR). In all datasets, infection rates were calculated using three different definitions for septic reoperation (Table 4) and the rates were compared to those achieved using the definitions used in the statistical analysis (combined endpoint data). The results of sensitivity analysis are presented in (Table 4; see supplementary data). Combined endpoint data indicated the highest number of infections in all datasets studied, but the infection rate was not significantly different from that detected with the endpoint data of HDR or FAR data only in any of the datasets. In all datasets HDR and FAR endpoint data yielded similar infection rates. Very few infections (overall infection rate (0.07% (0.07–0.48)) were detected after the index operations registered by the HDR only.

## Discussion

In this study we reviewed a large series of knee arthroplasties performed in 1997–2003. The register-based materials provided good coverage of the target population; thus, our study is a good representation of the current state of knee arthroplasty in Finland. We found an overall incidence of reoperations performed due to infection of 0.89%, which is similar to the rates of 0.86% and 0.80% that have recently been reported from specialized orthopedic institutions (Phillips et al. 2006, Jämsen et al. 2008). On a nationwide basis, a higher incidence of infections might have been expected. Comparison between the records of the 2 registers raised concern about their validity in the study of infected knee arthroplasties.

There are several possible explanations for the difference between the deep infection rates detected here and those reported previously. First, deep infections that fulfill appropriate diagnostic criteria but that are treated nonoperatively with antibiotic suppression or with a minor wound procedure remain undetected with our methodology. Such cases accounted for 57% of the cases that were registered by the Finnish Hospital Infection Surveillance Program SIRO but were missing from FAR in an earlier study (Kaisa Huotari, personal communication). Secondly, early infections that occur during the index hospitalization and which are treated with prosthesis retained are easily missed with register-based data that describe events as periods of hospitalization. Furthermore, because both the appropriate diagnosis and the surgical procedure code were required for a reoperation to be classified as septic, a number of prosthesis-related infections may have remained undetected due to absence of the infection diagnosis code in the HDR records. These methodological matters alone do not, however, explain the difference.

The data derived from the HDR confirmed our hypothesis that FAR does not reliably detect reoperations in which a new prosthesis is not implanted (such as debridements and resection arthroplasties) and therefore underestimates the infection rate. The effect of such ignored operations was most dramatic during the first 3 postoperative months. According to earlier FAR guidelines, revision arthroplasties performed within 4 postoperative months were considered to be postoperative complications. By now, this practice— which has led to underestimation of early failure rate in earlier cohorts—has been abandoned. Currently, early revisions are managed in the same manner as other revision arthroplasties and reporting activity probably plays a critical role. The specific reporting form for postoperative complications is rarely used, and complications are most likely registered if they lead to revision total knee arthroplasty (FAR covered 91% of these operations).

Similar problems have been reported previously in Sweden, where most of the reoperations that were not registered routinely in the Swedish Knee Arthroplasty Register were: extraction of prosthesis, patellar revision, partial exchange arthroplasty, arthrodesis, and amputation (Robertsson et al. 1999). In Norway, only 62% of the removals of knee prostheses were registered in the national arthroplasty register (Espehaug et al. 2006). This leads to overestimation of the prosthesis survival.

Although we did not specifically evaluate the validity of the HDR data, it seems that it is not without problems either, especially regarding insufficient use of infection diagnosis codes. This idea is supported by earlier literature stating that retrospective review of administrative databases lacks sensitivity (Romano et al. 2002, Curtis et al. 2004, Sherman et al. 2006) and on the other hand may include false positive entries (Romano et al. 2002, Sherman et al. 2006). Furthermore, reporting activity may dramatically bias comparisons between hospitals, as demonstrated by a study on complications after elective lumbar discectomy (Romano et al. 2002) where the sensitivity of administrative register data was lowest in hospitals with lower than expected complication rates, and at least half of the difference in complication rates seemed to be attributable to the differences in reporting activity only.

Despite the weaknesses discussed above, the registers were found to be fairly concordant. Most reoperations that were considered septic in the HDR were classified similarly in FAR and the registers yielded similar reoperation rates. Even though microbiological confirmation of infections was not available to us, it is likely that the infections recorded represented true cases as they led the orthopedic surgeon to proceed with a surgical intervention. Thus, the present materials can be used in the analysis of time trends and of relative differences between the 3 operation types.

Over the observation period, considerable changes were seen in several demographic variables and treatment protocols (Table 2). Decrease in the proportion of patients with rheumatoid arthritis, growth in the number of primary UKAs, shortening of hospitalization time, and increased popularity of antibiotic-impregnated cement would have been expected to result in fewer septic reoperations. The 1-year infection rates did not improve, however. The epidemic of obesity and diabetes, which increases the risk of postoperative infections (Peersman et al. 2001, Jämsen et al. 2008) and is common in patients undergoing TKA, could provide one explanation for such adverse development, but this hypothesis cannot be confirmed with the present register-based materials.

Potential bias in our study may be the methodology used to link the knee-level data of FAR to the HDR data on hospitalizations. Unless reoperations recorded in HDR could be first linked to a reoperation registered in FAR, the reoperations were linked to the most recent preceding index operation. This practice (which assumes that septic complications occur relatively early), was based on previous clinical and registerbased reports (Heck et al. 1998, Fehring et al. 2001, Sharkey et al. 2002, Phillips et al. 2006). It is possible that this method resulted in inclusion of reoperations that actually involved the contralateral knee or even some other joint, and thereby led to overestimation of the early failure rate. The problem of contradictory or missing data could have been resolved by reviewing data on patients’ other arthroplasties and also patient records. Such review, however, was not performed as our study was not designed to validate the data of FAR and HDR.

Readily available population-based databases with good coverage of the population of interest and a high number of cases registered, such as FAR and HDR in our study, appear to be an appealing source of data for volume-outcome and quality-of-care analyses; in fact, they are necessary to ensure sufficient statistical power (Guller 2006). Our findings suggest that FAR lacks sensitivity in detecting postoperative infections and therefore underestimates the numbers of such complications. Because the treatment approach affects the accuracy of detecting infections, the comparisons of infection rates derived from these data sources may be confounded by local differences in treatment practices and patient case-mix, as well as changes in treatment protocols over time. Unconfirmed data in FAR and HDR should not be used in comparisons between hospitals and between hospital districts until these issues have been considered and until the register data has been adequately validated.
